# Development and characterization of a novel poly(*N*-isopropylacrylamide)-based thermoresponsive photoink and its applications in DLP bioprinting†

**DOI:** 10.1039/d4tb00682h

**Published:** 2024-08-29

**Authors:** Kalindu D. C. Perera, Sophia M. Boiani, Alexandra K. Vasta, Katherine J. Messenger, Sabrina Delva, Jyothi U. Menon

**Affiliations:** a Department of Biomedical and Pharmaceutical Sciences, College of Pharmacy, University of Rhode Island Kingston RI 02881 USA jmenon@uri.edu; b Department of Chemical Engineering, College of Engineering, University of Rhode Island Kingston RI 02881 USA; c Department of Biomedical Engineering, College of Engineering, University of Rhode Island Kingston RI 02881 USA

## Abstract

The field of 3-dimensional (3D) bioprinting has significantly expanded capabilities in producing precision-engineered hydrogel constructs, and recent years have seen the development of various stimuli-responsive bio- and photoinks. There is, however, a distinct lack of digital light processing (DLP)-compatible photoinks with thermoresponsivity. To remedy this, this work focuses on formulating and optimizing a versatile ink for DLP printing of thermoresponsive hydrogels, with numerous potential applications in tissue engineering, drug delivery, and adjacent biomedical fields. Photoink optimization was carried out using a multifactorial study design. The optimized photoink yielded crosslinked hydrogels with strong variations in hydrophobicity (contact angles of 44.4° <LCST, 71.0° >LCST), indicating marked thermoresponsivity. Mechanical- and rheological characterization of the printed hydrogels showed significant changes above the LCST: storage- and loss moduli both increased and loss tangent and compressive modulus decreased above this temperature (*P* ≤ 0.01). The highly cytocompatible hydrogel microwell arrays yielded both single- and multilayer spheroids with human dermal fibroblasts (HDFs) and HeLa cells successfully. Evaluation of the release of encapsulated model macro- (bovine serum albumin, BSA) and small molecule (rhodamine B) drugs in a buffer solution showed an interestingly inverted thermoresponsive release profile with >80% release at room temperature and about 50–60% release above the gels’ LCST. All told, the optimized ink holds great promise for multiple biomedical applications including precise and high-resolution fabrication of complex tissue structures, development of smart drug delivery systems and 3D cell culture.

## Abstract 2

Biomedical applications of the 3D-printed thermoresponsive hydrogels developed using our DLP-compatible versatile photoink.

## Graphical abstract

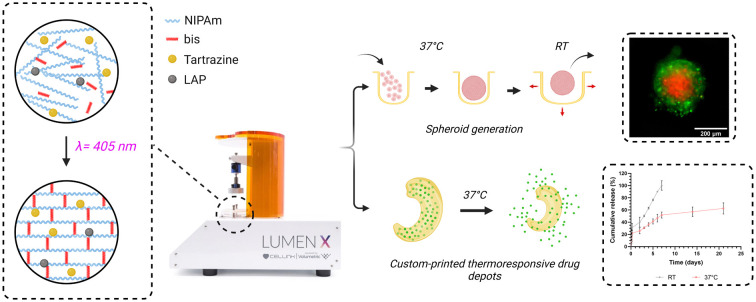

## Introduction

The field of 3D bioprinting – like 3D printing in general – is one that owes much to the development of stereolithography in the 1980s. This allowed the field of 3D bioprinting to blossom, with the Wake Forest Baptist Medical Center opening a new front in regenerative medicine with the work of Dr. Atala and colleagues in the late 1990s heading into the new millennium.^1–3^ These early attempts utilized simple droplet-based methods to print various tissue analogues. Digital light processing (DLP) as a concept was developed in 1987 building on stereolithography (SLA), but DLP based 3D bioprinting has only recently come to the forefront of the field of tissue engineering, owing to its ability to print complex shapes with features such as void spaces quickly and efficiently.^4,5^ DLP builds upon SLA, adding a digital micromirror device (DMD) that works by using thousands of tiny mirrors that can move in two dimensions and switch on and off at a rate of thousands of times a second.^6,7^ While SLA works by using a single ultraviolet (UV) laser beam to photo-crosslink/solidify a layer piecemeal, this DMD can essentially ‘project’ or ‘map’ the entire topography of a single layer (*via* the use of voxels, the 3D equivalent of a pixel) of the 3D file being printed upon the print bed containing liquid resin or a polymer solution.^6,7^ This allows an entire layer to be crosslinked/solidified/printed at once, drastically decreasing print times and increasing efficiency relative to SLA.^6–8^ In recent years, several different biopolymers such as polyethylene glycol dimethacrylate/-diacrylate (PEGDMA/PEGDA), silk fibroin, gelatin methacrylate (GelMA) and acrylated forms of hyaluronic acid have been used to develop DLP-printed constructs for a variety of biomedical applications.^9–13^

Poly(*N*-isopropylacrylamide) (PNIPAm) is a thermoresponsive and cytocompatible polymer that has been the subject of study since the 1950s demonstrating the ability to play a role in a wide range of applications, including drug delivery, sensors, soft robotics, tissue engineering, separation processes and more.^14–17^ NIPAm monomers are usually co-polymerized with *N*,*N*′-methylenebisacrylamide (bis) through free radical polymerization to produce PNIPAm.^14,17–20^ The polymer and hydrogel constructs made thereof are characterized by a lower critical solution temperature (LCST; usually around 32 °C^14,16,17,19^), at which a reversible phase transition takes place at the molecular level within the polymer. PNIPAm contains both amide- and isopropyl functional groups: at temperatures below the LCST, the hydrophilic amide group forms hydrogen bonds with water molecules, which in turn forms H-bonds with more water molecules.^18^ This causes a net inflow of water into the polymer matrix, and an exposure of the polymer's hydrophilic portion, resulting in swelling and hydrophilicity.^18^ At temperatures above the LCST, the amide groups tend to form H-bonds between themselves, causing the hydrophobic isopropyl groups to dominate, repel water and dictate the polymer's properties: this leads to hydrogel shrinking and hydrophobicity.^18^ This phase transition underpins the thermoresponsive behaviour of this polymer.

We have previously reported the development of PNIPAm-based hydrogel microwell arrays for the generation of 3D spheroids.^21^ This formulation involved chemical crosslinking, using tetramethylethylenediamine (TEMED)-ammonium persulfate (APS) crosslinking of acrylamide monomers: the formulation was poured into a custom-designed hard polymer negative micromold, and allowed to set overnight. These microwell arrays were used successfully to generate viable 3D cell spheroids using a variety of cancer and fibroblast cell lines. We now report a more streamlined approach for the generation of these microwell arrays which overcomes some of the shortcomings of the earlier method including potential cell toxicity of the APS/TEMED initiator-accelerator pair and long detachment times from the mold (about 5 to 14 days). We attempted to first print the thermoresponsive microwell arrays directly on a DLP-based bioprinter, later transitioning to the formulation of a photoink for a wider range of thermoresponsive applications.

Thus, we report here the successful optimization, formulation, and characterization of a novel DLP bioprinter-compatible photoink using NIPAm as the primary component and minimal copolymerization agents. We show here that the ink is capable of printing multiple complex structures with fine features and have robust mechanical and thermoresponsive properties for the intended *in vitro* work. We present this formulation as a platform to use for a multitude of possible applications in which thermoresponsivity may aid in modelling, drug delivery and tissue engineering.

## Experimental

### Materials

NIPAm, bis, LAP, phosphate buffered saline (PBS) and Dulbecco's modified Eagle medium (DMEM) were purchased from Sigma-Aldrich (St. Louis MO, USA). All cells were cultured in DMEM supplemented with 10% fetal bovine serum (FBS) and 1% penicillin–streptomycin purchased from Thermo Fisher Scientific (Waltham MA, USA). Tartrazine, bovine serum albumin (BSA), multicolour cell staining kit and bicinchoninic acid (BCA) assay kit were similarly purchased from Thermo Fisher. LIVE/DEAD™ Viability/Cytotoxicity Kit was purchased from Invitrogen/ThermoFisher, and a WST-8 cell proliferation assay kit was purchased from Cayman Chemical (Ann Arbor MA, USA). All kits were used following manufacturer protocols. Silicon chip specimen supports (wafers) were purchased from Ted Pella (Redding CA, USA) for electron microscopy. HeLa cervical cancer cells and human dermal fibroblasts (HDFs) were purchased from the American type culture collection (ATCC; Manassas VA, USA).

### Photoink formulation

Initial printing was based on a modified version of our previously-established formulation.^21^ In brief, NIPAm, bis, LAP and tartrazine were mixed in deionized water (DIW) at concentrations of 200 mg mL^−1^, 8 mg mL^−1^, 2 mg mL^−1^, and 0.5 mg mL^−1^ respectively. This was subjected to several rounds of full factorial design optimization (see ESI,† Tables S1–S3), exploring both formulation and print parameters. The first of these rounds of optimization focused on formulation parameters, being a 4-factor, 2-level design: NIPAm (at 100- & 200 mg mL^−1^), bis (4- & 8 mg mL^−1^), LAP (1- & 2 mg mL^−1^) and tartrazine (0.1- & 0.25 mg mL^−1^). The second was centered on print parameters, being a 3-factor, 2-level design: power (at 36- & 44%), layer exposure time (16- & 20 s) and base layer exposure factor (2- & 4×). The third and final round of optimization refocused on formulation parameters, again *via* a 4 × 2 design: NIPAm (at 100- & 150 mg mL^−1^), bis (8- & 16 mg mL^−1^), LAP (2- & 3 mg mL^−1^) and tartrazine (0.25- & 0.5 mg mL^−1^). Full factorial experiments were designed on Minitab v20.4 (Minitab LLC, USA). From these candidate formulations, a final formulation was selected for further characterization and experimentation. This consisted of 150 mg mL^−1^ NIPAm, 8 mg mL^−1^ bis, 2 mg mL^−1^ LAP and 0.5 mg mL^−1^ tartrazine.

**Fig. 1:**
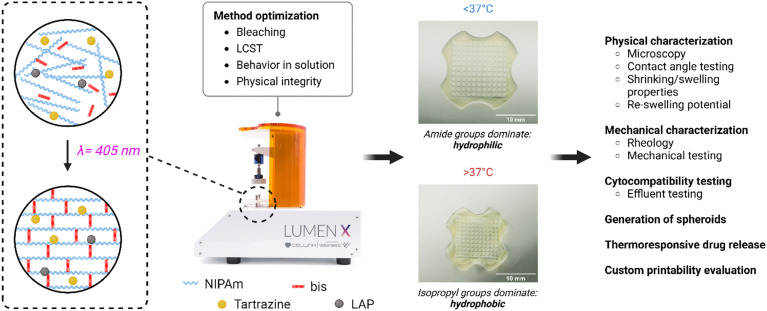
Summary of gel-making process. The goal of the work was to produce hydrogels for several biomedical applications at high print fidelity and an LCST of approx. 37 °C. Optimization was done to eliminate issues such as ‘bleaching’.

This process, from inception to final optimization, is summarized in Fig. 1.

### Hydrogel printing

**Fig. 2:**
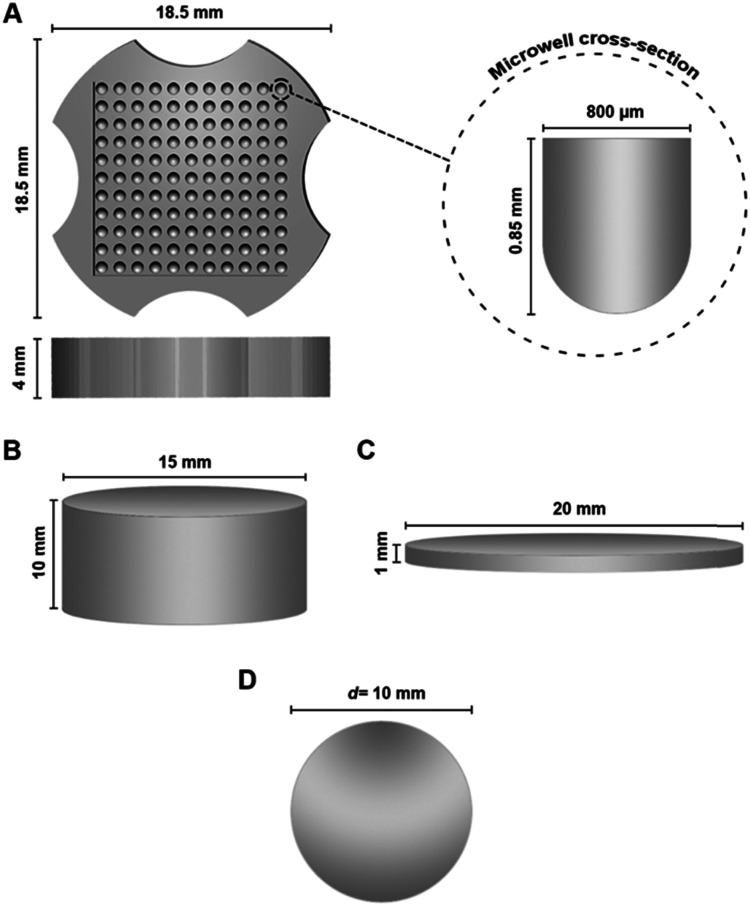
STL files of the different structures printed for physical- and *in vitro* characterization. (A) 12 × 12 array of microwells used for spheroid generation, with wells having a diameter of approx. 800 μm at the time of printing/at room temperature; (B) cylinder used to perform LCST and contact angle testing; (C) discs used to perform rheology/compression testing; (D) spheres used to evaluate drug release and *in vitro* cytotoxicity. Figures not to scale.

Printing was performed using the LumenX+ DLP bioprinter (Cellink, USA) using ink that was pre-cooled to 4 °C. All in-house STL files (Fig. 2) were designed on Microsoft 3D Builder; different structures such as microwell arrays, spheres and cylinders were printed using the bioink according to the requirements/specifications of the different characterization experiments conducted. The initial formulation (unoptimized) was printed with printer settings as follows: 44% power, 18 s exposure time and a ×4 base exposure factor. All printing subsequent to optimization was performed with settings as follows: 44% power, 18 s-layer exposure time and a ×5 base layer exposure factor. Complete prints were gently removed from the printhead following conclusion of printing and washed in DIW on a rocking shaker at room temperature (RT) for 6–8 hours (with replacement of water every hour). All printed structures were visually confirmed to be fully washed of photoabsorber prior to subsequent studies/assays unless otherwise noted. Washing was deemed complete when there was no visible leaching of tartrazine into the DIW used for washing (*i.e.* no visible yellow coloration).

### Physical characterization

#### Microscopy

Both scanning electron microscopy (SEM) and EVOS (Life Technologies, USA) light microscopy were carried out for the purposes of determining surface morphology and microwell sizing of printed hydrogels. For SEM, hydrogels were subjected to dehydration by sequential immersion within a series of ethanol–phosphate buffered saline (PBS) solutions ranging from 50 to 100% ethanol by volume. Samples were left to dry overnight, followed by mounting on SEM stubs, gold sputtering and imaging on a Zeiss Sigma VP field emission scanning electron microscope (Carl Zeiss, Germany). All imaging was carried out at an acceleration voltage of 10 kV, using the SE2 detector. All EVOS microscopy images collected herein were subjected to sizing and scale bar re-addition using ImageJ software (v1.53e) for clarity, as were SEM micrographs.

#### Lower critical solution temperature (LCST)

LCST of hydrogels printed using all tested formulations were determined *via* absorbance measurements using a Synergy H1 multi-mode reader (Biotek, USA) (and accompanying Gen5 software package, v3.09.07). The LCST was defined as the temperature producing half of total increase in absorbance (*i.e.* the midpoint of a linear OD *vs.* temperature graph). For this purpose, cylindrical hydrogels with dimensions of 15 × 10 mm (Fig. 2B) were printed using each of the full factorial formulations. These were placed in clear-bottomed 12-well plates (*n* = 3) and heated using the reader's internal thermostat from 25 to 45 °C at a ramp rate of 0.4 °C min^−1^; absorbance at *λ* = 500 nm was measured at 2 °C degree intervals. Data was subjected to derivatization and graphed, with temperature as the independent variable and second derivative of the absorbance value as the dependent variable: LCST was found by determining the point at which the graph intersected with the *x*-axis. Graphing and LCST identification were performed on OriginLab (v10.0.0.154; OriginLab, USA).

#### Shrinking behaviour

Hydrogels were immersed directly in a temperature-controlled water bath containing DIW at 20 °C, to allow for swelling. The temperature of the bath was increased gradually in 5 °C increments from 20 to 45 °C, with 10 minutes’ equilibration at each temperature. Once fully equilibrated, gels were blotted dry carefully with filter paper and weighed at each temperature point. Gels were subjected to lyophilization, and their weights were recorded again. The shrinking ratio (SR) was calculated by means of eqn (1), wherein *W*_s_ is the weight of the sample at each time point and *W*_d_ is the weight of the dry, lyophilized sample.1
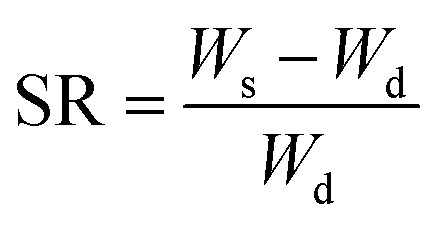


#### Swelling behavior

Lyophilized hydrogels were weighed, then immersed in DIW at RT. Swollen gels were then weighed at predetermined time points after blotting dry carefully with filter paper. Swelling ratio was calculated using eqn (1), wherein *W*_s_ is the weight of the swollen sample at each timepoint and *W*_d_ is the weight of the dry, lyophilized sample.

#### De-swelling kinetics

The reversible phase transition behavior of the printed hydrogels was confirmed using de-swelling and re-swelling studies. Lyophilized hydrogels were weighed, then immersed in DIW at RT for 5 days. The swollen gels were weighed again and allowed to de-swell in DIW at 45 °C and weighed at predetermined time points (every ten minutes from 0 to 2 hours, and every hour thereafter until the 4-hour mark) after careful blotting with filter paper. The degree of water retention (% WR) was calculated using eqn (2), wherein *W*_t_ is the weight of the sample at each timepoint, *W*_d_ is the weight of the dry, lyophilized sample, and *W*_e_ is the weight of the equilibrium swollen sample at RT (25 °C).2
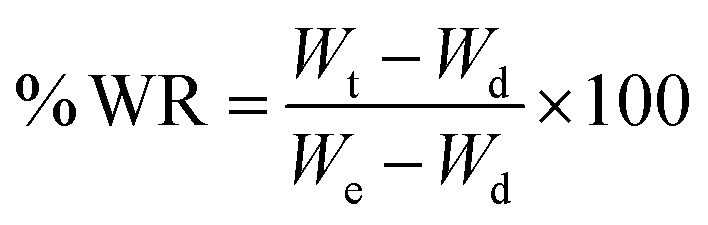


#### Re-swelling degree

For this study, lyophilized hydrogels were immersed in DIW at 45 °C and allowed to equilibrate for 2 h. They were then moved to DIW at 25 °C and weighed at predetermined timepoints (see de-swelling kinetics) following careful blotting with filter paper. The degree of reswelling (% RD) was calculated using eqn (3), wherein *W*_r_ is the weight of the sample at each timepoint, while *W*_e_ is the weight of the equilibrium swollen sample at 25 °C.3
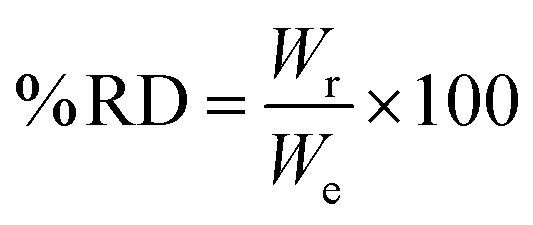


#### Contact angle measurement

Contact angle measurements were carried out to assess temperature-dependent changes in hydrophilicity in prints of the final formulation. Measurements were performed with a Krüss Drop Shape Analyzer S100 (Krüss, Germany) and accompanying ADVANCE software (v1.7.1.0), with a 200 μL drop volume (DIW). Fully washed cylindrical hydrogels were incubated for 2 h at either 4- or 37 °C prior to performing measurements. Images of contact angle and absolute contact angle values were obtained directly from the instrument camera and ADVANCE software.

#### Dynamic mechanical analysis

A Discovery HR30 rheometer (TA Instruments, USA) was used to perform all testing related to the mechanical properties of hydrogels printed using the final chosen formulation.

Dynamic rheology and compressive stress testing were performed on 20 × 1 mm discs (Fig. 2C) at three fixed temperatures (*n* = 3 each): 4-, 25- and 37 °C, due to the thermoresponsive shrinking of the gels making a temperature sweep on fixed geometries impractical. Thus, a 20 mm parallel plate geometry was utilized for temperatures <LCST, while an 8 mm plate setup used for gels at >LCST. A strain sweep was conducted to determine the linear viscoelastic region of the final chosen formulation, at 10 rad s^−1^, over a range of 0.01 to 100% strain. Elastic- (*G*′) and viscous (*G*′′) moduli as well as complex viscosity were determined *via* a frequency sweep measurement within the linear viscoelastic region of the gel at 0.1% strain amplitude and angular frequencies from 100 to 0.1 rad s^−1^. Compressive stress testing was done at a constant linear rate of 10 μm s^−1^ on the same apparatus at all three temperatures.

All data analysis was performed on TA Instruments’ TRIOS software (v5.1.1).

#### Print limit evaluation

A number of hydrogels with surface and sub-surface features were printed using the final formulation in order to establish limits of printability. EVOS microscopy and macro photography were utilized to visualize and compare planned and actual sizes of these features.

### *In vitro* studies

For all cell-based *in vitro* experiments, washed hydrogels were incubated in PBS at 37 °C for 30 min, following which they were sterilized by brief incubation (10–15 s) in 70% ethanol. The gels were then transferred immediately to a 6-well plate, and sterile complete DMEM (10% FBS, 1% pen/strep) was added to the wells. Equilibration was carried out overnight by incubating the gels in standard culture conditions (37 °C, 5% CO_2_). Equilibration medium was removed immediately prior to cell culture experiments.

#### Formulation cytocompatibility

Spherical hydrogels with a diameter of 10 mm (Fig. 2D) were immersed in 1 mL complete DMEM for a period of 1, 3, 5 and 7 days at standard culture conditions. At each timepoint, spent media was collected and stored at −20 °C until required. HeLa cells seeded at a density of 10 000 cells per well were then treated with this spent media at standard culture conditions for 24 h. WST-8 assay was then carried out per manufacturer's instructions to determine potential cytotoxicity of leached hydrogel components on these cells. All viability results were calculated relative to untreated controls. These results were corroborated using live/dead staining.

#### Spheroid generation and characterization

Microwell arrays (Fig. 2A) were seeded using suspensions of human dermal fibroblasts (HDF) at 36 and 72 × 10^5^ cells per mL on microwell arrays (100 μL per gel). Cells were stained with Dil prior to seeding, following manufacturer instructions. Seeded gels were allowed to rest/incubate at standard culture conditions for 15 minutes to allow cells to settle in microwells. Complete culture media was then reintroduced to the wells containing the arrays, ensuring the arrays themselves were immersed within it. These were then left to incubate for 24 hours at standard culture conditions, following which visual and microscopic (*via* EVOS imaging) observations were made for the size and morphology of formed spheroids. Gels were maintained for up to 7 days (with media replenishment every 48 hours) post-seeding, with observations made as outlined above on days 1, 3, 5 and 7 post-seeding.

ImageJ (v1.53e) was used to measure the circularity and volume of spheroids subsequent to EVOS imaging. The software's built in circularity function was used for the former; volume was calculated using the spheroids’ Feret diameter.

#### Generation of core–shell spheroids

The co-culture of multiple cell types as core–shell spheroids within the microwell arrays was evaluated next. HeLa cells at 36 × 10^5^ cells per mL (100 μL per gel) seeding density, were seeded onto microwell arrays previously seeded with HDF at 72 × 10^5^ cells per mL density, following the method outlined in Spheroid generation and characterization. Cells were stained with DiO prior to seeding, following manufacturer instructions. Resultant microwell arrays were imaged on the EVOS microscope. Overlaid compositing of red and green channel images was carried out on ImageJ.

#### Drug release study using 3D printed PNIPAm drug depots

The release behaviour of two model drugs, BSA and rhodamine B, at two temperatures (room temperature and 37 °C) was assessed in PBS at pH 7.4. Two different approaches for drug loading were implemented for these studies; the BSA was added directly to the photoink during printing while rhodamine B was loaded into the hydrogel post printing.

To produce BSA-loaded gels, spherical hydrogels with a diameter of 10 mm were printed using the PNIPAm photoink supplemented with 1 mg mL^−1^ BSA and used without washing (tartrazine was determined not to interfere with the BCA assay). These were then immersed individually in 10 mL PBS each and left to shake on an orbital shaker at room temperature and at 37 °C (*n* = 3, both groups). Sampling was carried out at predetermined intervals: 200 μL aliquots of the PBS were drawn out and replaced with an equal volume of fresh PBS; withdrawn aliquots were stored at −20 °C until further analysis. BCA assays were used according to manufacturers’ instructions to determine the BSA release kinetics from the hydrogels at both temperatures.

To produce rhodamine B-loaded gels, spherical hydrogels were printed, washed for 6–8 hours as outlined previously, and immersed in a solution of rhodamine B in DIW at 10 mg mL^−1^ and left to equilibrate for 48 hours at 4 °C on a rocker–shaker, following which the spheres were washed by rinsing briefly with DIW, and then immersed in DIW for a further 12 hours at 4 °C on the shaker to remove any dye loosely bound/not fully incorporated into the gel matrix. These washed gels were then immersed in PBS and incubated at two temperatures, and sampling carried out as outlined above. Quantification of released dye was performed by preparing a standard curve of the dye in PBS and measuring absorbance of these and sampled aliquots (as well as wash solutions) at 554 nm using a plate reader.

Finally, several non-standard structures such as a miniature human kidney and liver, as well as an enlarged model of a bacteriophage virus and a hollow block with a miniature human carotid were printed using the final chosen formulation to demonstrate the range of the photoink's applicability.

### Statistical analysis

All statistical analysis was performed on GraphPad Prism 9.0.0 (GraphPad Software Inc., USA) unless otherwise noted. Graphing was performed on GraphPad Prism 9.0.0 or OriginLab (v10.0.0.154; OriginLab, USA).

## Results & discussion

### Formulation optimization

As part of this investigation, our early work attempted a direct translation of our previously reported work^21^ into the DLP printing space, maintaining the concentrations of NIPAm and bis of that formulation while exploring the effect of multiple concentrations of LAP and tartrazine. This, however, produced inconsistently opaque (“bleached”) printed structures that reduced their potential for optically-sensitive work such as imaging, and made lower critical solution temperature (LCST)-determination impossible (see Fig. S1 in ESI†). A 4 × 2 factorial experiment was then carried out for the purposes of optimization, with bleaching as the main dependent variable of interest; the LCSTs of unbleached or partially-bleached hydrogels were also measured. The results of this work are presented in the ESI† (Table S1). This round of optimization yielded many formulations that did not produce bleached prints; formulations that did produce bleaching pointed to the concentrations of both NIPAm and bis as being a putative cause: high concentrations of both appeared to cause consistent bleaching. In addition, the lower concentration of NIPAm used (100 mg mL^−1^) herein tended to produce gels that were insufficiently rigid, to a degree unsuited to handling and cell culture.

A second 3 × 2 factorial experiment was carried out (Table S2, ESI†), seeking to establish whether printer parameters influenced the bleaching effect: the original formulation from our previous work utilized herein. This factorial experiment produced highly inconsistent bleaching (producing replicates of the same factorial run that were bleached either fully or in part) and was thus halted part-way: this appeared to point to print factors not playing any notable role in the bleaching. A third 4 × 2 factorial experiment was carried out (Table S3, ESI†) with formulation component concentrations as variables again. This served to confirm that bleaching was proportional to the concentration of bis used and yielded several formulations that possessed LCSTs approaching the desired one of 37 °C. Hydrogels printed using these formulations were incubated in either PBS or complete DMEM in standard culture conditions: formulations were excluded based on any marked distortion to the structure of the printed gels. Interestingly, gels appeared to hold their shape better in PBS than in DMEM, with most formulations/gels exhibiting more marked inward ‘curling’ in DMEM. This may possibly be an effect of osmolality: DMEM possesses a osmolality of 330–360 mOsm kg^−1^,^22,23^ compared to the approximately 300 mOsm kg^−1^ figure quoted for PBS.^24–26^ The stronger curling effect observed in DMEM may thus be a result of both the polymer's inherent thermoresponsivity and the osmotic pressure exerted by the medium causing higher/more rapid shrinkage of printed hydrogels. In this manner, formulation 7 from full factorial experiment 3 was selected for further study in this work. This final optimized formulation was found to yield hydrogels with a LCST of 36.8 °C.

It must be noted that these cross-linked hydrogels did not appear to undergo any noticeable degradation in the cell culture medium up to a period of 7 days in this study. Gels were observed to be unchanged after 14 days in previous work by our group, and indeed, PNIPAm-based gels have been used without degradation for up to 70 days by other groups.^21,27^

### Preliminary physical characterization

The successful printing of hydrogels using this photoink relies on the interplay between its components: NIPAm, bis, LAP and tartrazine. LAP is a cytocompatible type I photoinitiator, undergoing photoinduced, homolytic cleavage into constituent benzylphosphinate and trimethylbenzene free radicals.^28–31^ The ensuing free radical-driven copolymerization of NIPAm and bis is one that has been well-characterized and -exploited in the past.^32–34^ The role of tartrazine as the photoabsorber is to improve print resolution and guarantee print fidelity by absorbing light scattered at the photoink–hydrogel interface (*i.e.* as the print is in progress) and light penetrating beyond the printer's depth of focus.^35,36^ In both cases, this prevents over-/uncontrolled curing (particularly of void spaces such as the microwells in Fig. 2A).^35,36^

**Fig. 3:**
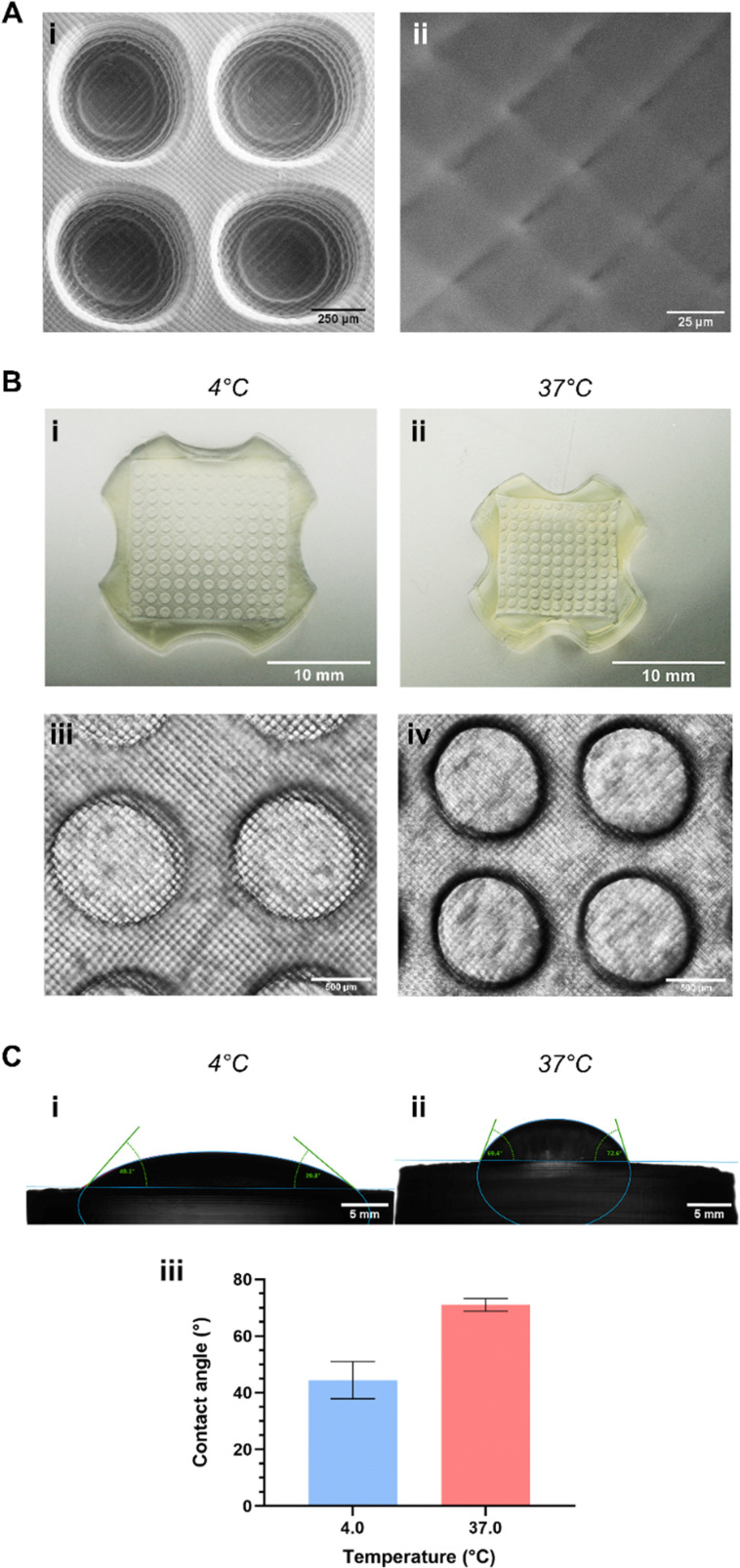
Structural characteristics of hydrogels printed using the final chosen formulation. (A) Scanning electron micrographs of microwells (i), with all printed structures exhibiting the cross-hatch pattern (ii) caused by printer voxels; (B) images of whole printed microwell arrays and EVOS microscopy images of microwells below (i) and (iii) and above (ii) and (iv) the formulation's LCST, showing hydrogel propensity to shrink beyond the LCST; (C) angle of contact created by DI water on printed hydrogel discs: (i) and (ii) images, and (iii) graphical representation, showing clear switch in hydrophilicity at the LCST.

Hydrogels printed with the chosen final formulation of photoink were evaluated for several physical characteristics. Hallmarks of voxel-based printing were visible within the microstructure of printed hydrogels, with both SEM imaging (Fig. 3A) and EVOS microscopy (Fig. 3B-iii and iv) showing the resultant cross-hatch pattern clearly. SEM imaging in particular showed that the surface of these gels was not uniformly smooth, although the cross-hatch does not produce markedly deep grooves on the surface, certainly not deep enough to prove any great obstacle to spheroid- or cell sheet-based *in vitro* work.

The gels readily exhibited thermoresponsive properties at temperatures above the formulation's LCST: Fig. 3B and C illustrate this phenomenon clearly using printed microwell arrays. The bulk of the gel tended to swell as they were cooled and shrank noticeably as the temperature exceeded the formulation's LCST (Fig. 3B-i and ii): this was reflected in the microstructure of the printed gels, with microwells swelling and shrinking in a similar manner (Fig. 3B-iii and iv). Microwells were found to swell to a maximal diameter of approx. 1100 μm at 4 °C and shrink to a diameter of approx. 550 μm at 37 °C: indeed, the STL files for printing the microwell arrays described herein were designed to take this phenomenon into account based on our previous work with this polymer. Microwells were designed such that when shrunk, they would produce cell spheroids of a size known to induce the formation of a quiescent and necrotic core, mimicking tumors *in vivo*.^37,38^ Thermoresponsive swelling/shrinking of PNIPAm-based hydrogels (an effect of the polymer's LCST-induced phase transition) has been well-documented, as has the characteristic hydrophilic-to-hydrophobic switch during this same transition.^19,20,39^ This property too was apparent in the printed hydrogels: contact angle studies (Fig. 3C) showed that gels were hydrophilic at temperatures below their LCST (mean contact angle *θ* of 44.4 ± 6.6°) and considerably less so above this temperature (mean *θ* of 71.0 ± 2.3°). While this latter contact angle does not indicate ‘true’ or classical hydrophobicity (which requires *θ* > 90° (ref. 40)), it nevertheless indicates a relatively more hydrophobic hydrogel exists above the formulation's LCST. Interestingly, this is a notably more dramatic shift in hydrophilicity from that observed in our previously-published work.^21^

The limits of the formulation – in terms of the smallest printable features – were evaluated using a variety of hydrogel structures with various surface and sub-surface features on the micron scale. One such trial is illustrated in the ESI,† Fig. S2. In the case of raised surface features, the limit for height was found to be as low as 40 μm, although the limit for width was 80 μm. Essentially, a ridge-like feature on the surface of a hydrogel would print along the *y*-axis at a length of 40 μm but could not resolve along the *x*-axis at lengths below 80 μm. Conversely, for recessed surface features, the limit appeared to be 100 μm. For subsurface/channel-like circular features (as shown in ESI,† Fig. S2), the limit was established to be 200 μm in diameter, as anything below this did not print successfully.

### Swelling and shrinking behaviour

**Fig. 4:**
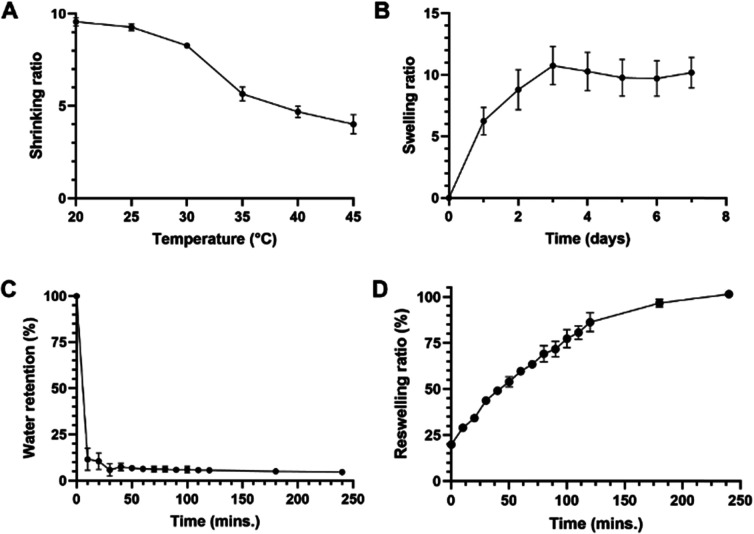
Swelling and shrinking characteristics of hydrogels printed using the final chosen formulation. (A) Shrinking study indicates slowing rate of shrinking at temperatures >35 °C; (B) swelling studies at 25 °C show a relatively plateaued swelling pattern after day 3; (C) de-swelling studies at 45 °C shows a rapid decrease in % water retention in the first 10 min., following which it remained relatively constant; (D) re-swelling studies at 25 °C shows complete re-swelling within 200–250 min. All experiments performed at *n* = 4.

To quantify and further explore the swelling/shrinking behaviour previously noted, hydrogels were subjected to several shrinking- and swelling-based studies. The gels demonstrated clear temperature-dependent shrinking: they underwent a 2.39-fold decrease in the shrinking ratio over the range of temperatures tested (Fig. 4A), although they may undergo further shrinkage, given that a plateauing effect is not observed up to the 45 °C mark. The swelling behaviour of dry hydrogels at 25 °C is shown in Fig. 4B: samples achieved maximum swelling on day 3 and then plateaued, leading to no significant change in weight from days 3 to 7. Nearly 60% of overall swelling appeared to have taken place within the first 24 hours. De-swelling studies (Fig. 4C) revealed that the loss of water from the hydrogel upon transfer from 25 to 45 °C was very rapid, with an 81% decrease in water content within the first 10 minutes (water content remaining stable thereafter). This photoink thus yields hydrogels with strong, rapid and reversible thermoresponsive behaviour. The ability of the hydrogels to re-swell was then assessed by transferring gels incubated at 45 °C to 25 °C, and results are presented in Fig. 4D. This showed that these gels were capable of re-swelling to almost their full weight after exposure to high temperatures beyond their LCSTs, returning to 99.9% of original weight within 240 minutes, showing a good degree of robustness for applications requiring multiple heating and cooling steps/cycles. The results of these studies indicate that the hydrogel produced by DLP printing of our chosen formulation of photoink undergo rapid (≤10 min) shrinking by a factor of approximately 1.7-fold at its LCST. In sum, these results are of great importance to tissue engineering applications such as spheroid- or cell sheet generation, where not only the change in hydrophilicity of the hydrogel is key, but also the shrinking/swelling properties: such changes facilitate either cell compaction (when shrunk) or facile detachment/removal (when swollen).

Overall, this further reinforces the much more noticeable switch in hydrophilicity at this formulation's LCST first noticed during initial contact angle studies. This may be attributed to two factors: firstly, the lack of acrylic acid (AA) within the ultrastructure of these hydrogels. AA is known to be strongly hydrophilic and is commonly used as a co-monomer in the formation of PNIPAm hydrogels alongside bis and TEMED (incorporated either randomly or as an interpenetrating network).^41,42^ Incorporation of AA into PNIPAm hydrogels has also been shown to reduce the thermoresponsivity of PNIPAm hydrogels.^42^ Taken together, the lack of AA within our formulation results in hydrogels with fewer hydrophilic groups (leading to gels losing water quicker) and a more unrestrained potential for thermoresponsivity, a property that the de-swelling kinetics in particular show quite clearly. Secondly, the thermoresponsivity of PNIPAm hydrogels is known to be affected by the cross-linking density.^43,44^ There has been no comprehensive study of the difference between chemical- and photo-crosslinking of PNIPAm in terms of the density of crosslinking, mesh size and other properties of the hydrogels produced by both techniques (which are in turn affected by a multitude of variables including reaction/exposure time, concentration of initiators and other formulation components). Cross-linking density has been shown to be a key factor in determining swelling/shrinking kinetics and even drug release from hydrogel matrices.^44–47^ It is possible, then, that the results seen in Fig. 4 may be a result of more dense cross-linking within the formulation presented herein, leading to quicker hydrogel collapse at temperatures above the LCST or recovery/expansion below it.

### Mechanical analysis: rheology and mechanical testing

**Fig. 5:**
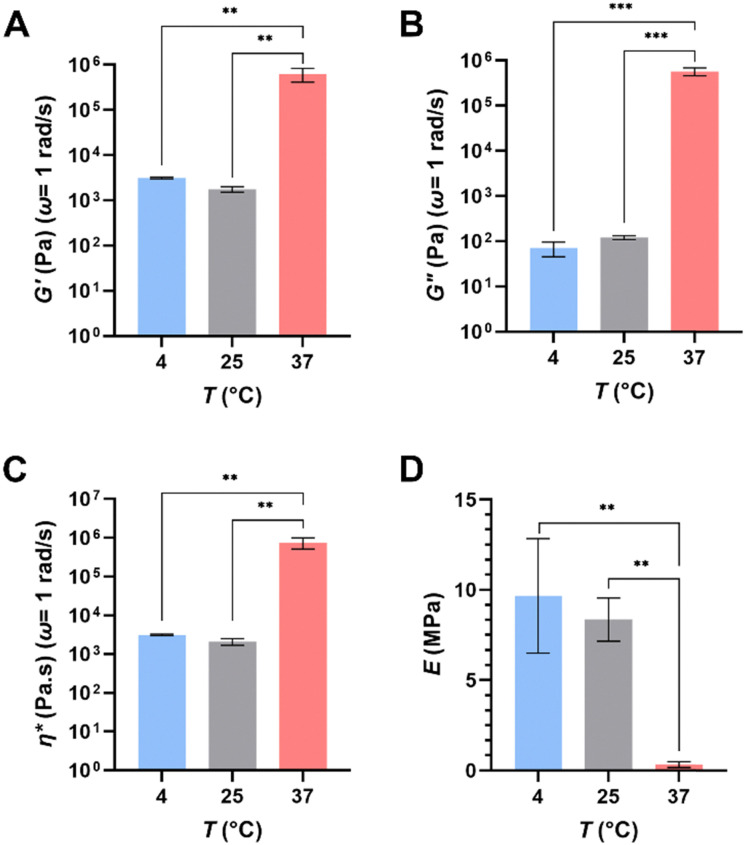
Results of rheological and compression testing of cross-linked hydrogels printed using the final chosen formulation. (A) Quantification of the storage moduli, *G*′, (B) loss moduli, *G*′′, (C) complex viscosity, η* and (D) compressive (Young's) moduli, *E*, of hydrogels at three different temperatures. All results reported for at least *n* = 3. ** *p* ≤ 0.01, *** *p* ≤ 0.001.

Bulk rheology testing was performed at three key temperatures: 4-, 25- and 37 °C, representing a low temperature extreme, ambient temperature in a laboratory setting and the typical temperature used in mammalian cell culture. Overall, the results of this work (summarized in Fig. 5; see Fig. S3, ESI† for mean trends of rheological parameters) indicate that the hydrogels exhibit temperature-dependent changes in their viscoelastic properties, significant particularly above their LCST. The storage modulus (*G*′) of the gels saw a significant decrease from 625.62 ± 211.61 kPa at 37 °C to 1.77 ± 0.24 kPa at 25 °C and 3.14 ± 0.15 kPa at 4 °C (Fig. 5A). The trend was reflected in their loss modulus (*G*′′) 575.00 ± 116.25 kPa to 0.121 ± 0.012 kPa to 0.071 ± 0.025 kPa in the same order; (Fig. 5B) and their complex viscosity (753 582.67 ± 242 048 Pa s to 2082.52 ± 417.15 Pa s to 3139.74 ± 146.48 Pa s in the same order; Fig. 5C) as well. Loss tangents (tan *δ*) calculated for 37-, 25- and 4 °C were 0.919, 0.0684, and 0.0226 respectively. Tan *δ* being <1 indicated the crosslinked gels tended to exhibit mainly elastic behaviour. Compression testing revealed the average Young's modulus of the hydrogels to be 9.66 ± 3.17 MPa, 8.35 ± 1.19 MPa and 0.33 ± 0.17 MPa at 4 °C, 25 °C, and 37 °C respectively (Fig. 5D). These results indicate that the hydrogel exhibits a significant decrease in stiffness and ductility at temperatures above the LCST. This can be attributed to the close packing of polymer chains above LCST, limiting their ability to move and twist around one another.

These trends are generally consistent with those previously reported for PNIPAm-based formulations, although it is difficult to carry out direct comparisons with formulations that involved different components or polymerization methods (*i.e.* chemical *vs.* light-driven). PNIPAm-containing hydrogels have been documented to exhibit more elastic behaviour below the LCST and more viscoelastic behaviour above it.^48^ Increases in *G*′ above the LCST and decreases below it was reported by a study investigating the mechanical properties of PNIPAm/cellulose nanofibril composite hydrogels, while increase in gel stiffness (attributed to interchain hydrophobic interactions) was reported by another group.^49,50^ A study comparing mechanical properties of PNIPAm in the presence of two different crosslinkers (one being bis) at various concentrations reported a similar pattern of results for storage- and loss moduli, as well as loss tangents.^51^ It is interesting to note that a number of authors have reported compressive moduli at various temperatures below the LCST of their hydrogels that are an order of magnitude lower than those reported herein.^52–56^ This could be due to higher crosslinking density in our hydrogels, the photopolymerization method employed, or a combination of both factors.

### Evaluation of cytocompatibility of hydrogel effluents

**Fig. 6:**
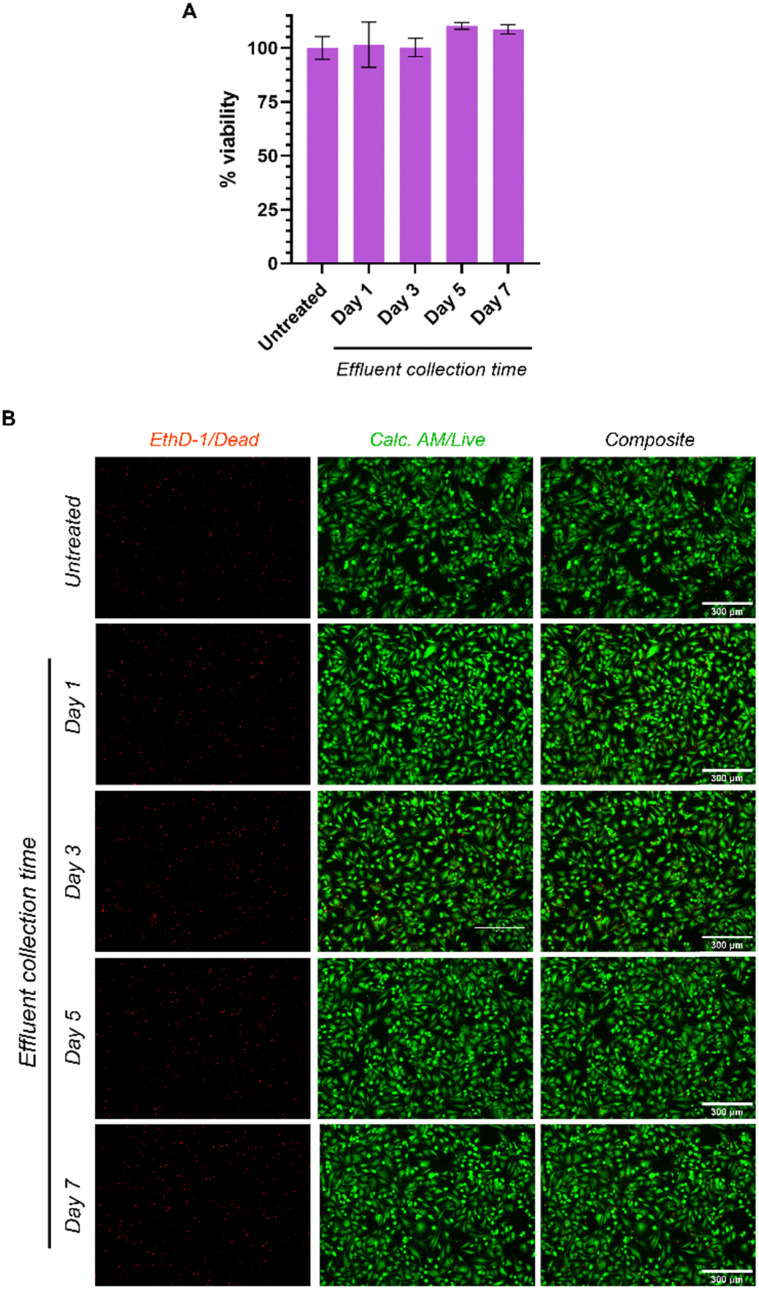
Effects of hydrogel effluent on the viability of HeLa cells, days 1–7, represented in the form of (A) WST-8 cell proliferation assay, and (B) EVOS fluorescence micrographs of live-dead staining of same, indicating no significant cell death up to day 7. All scale bars = 300 μm.

To test the cytocompatibility of effluents from the printed hydrogels, cell culture media pre-incubated with hydrogels for 1, 3, 5 and 7 days was added to HeLa cells, and cell viability evaluated after 24 hours of treatment. 100% viability or greater was observed in all treatment groups relative to the untreated controls (Fig. 6A). These findings were corroborated using live/dead staining, which showed that cells treated with the hydrogel effluents were viable (green) and no cell death (red) was observed (Fig. 6B). Our results confirm that a printed hydrogel does not leach out toxic by-products, which is in line with other reported cytocompatibility studies for PNIPAm-based hydrogels.^57–59^

### Spheroid generation and characterization

**Fig. 7:**
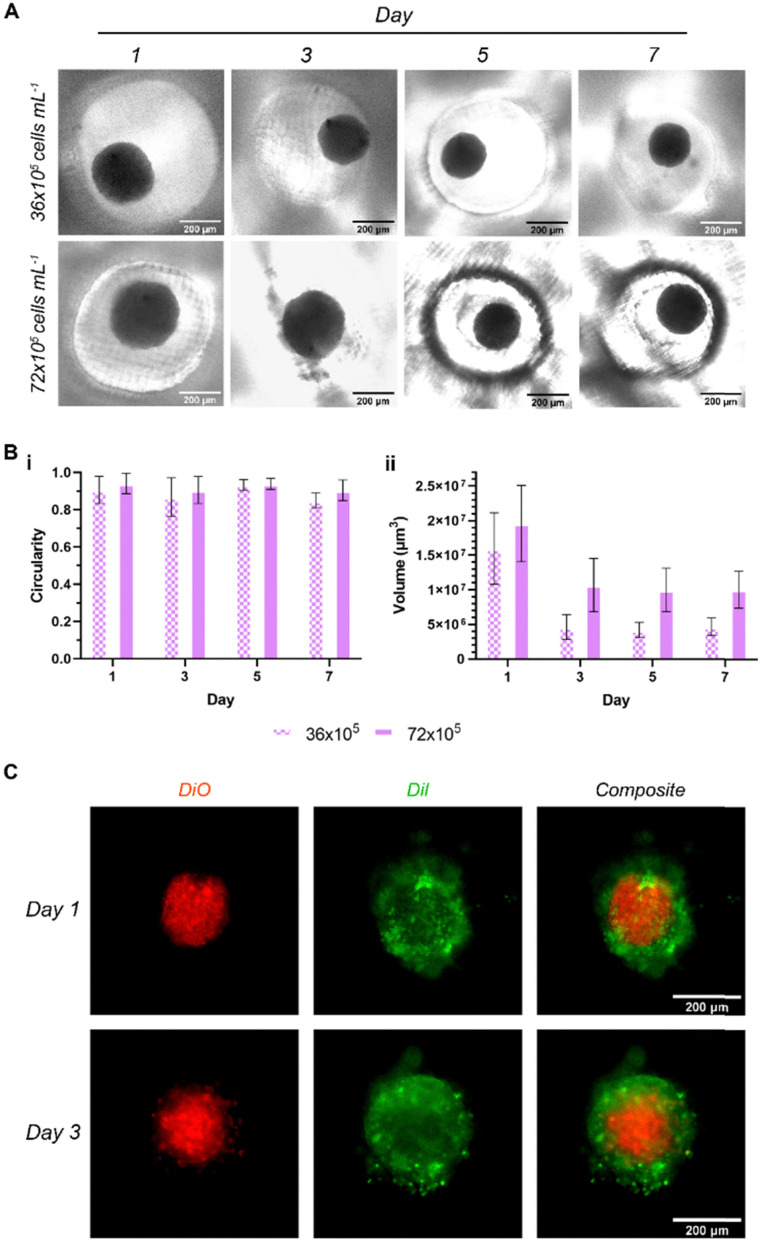
Visualization of spheroids and their characteristics. (A) EVOS micrographs of HDF monoculture spheroids over days 1–7 at two seeding densities, (B) quantification of (i) circularity and (ii) volume of HDF spheroids (*n* = 8), and (C) EVOS fluorescence micrographs of bilayer spheroids, days 1 and 3 (red/inner = HDF, green/outer = HeLa). All scale bars = 200 μm. Further images available in ESI,† Fig. S4.

HDFs were seeded at two seeding densities to assess the ability of the microwell arrays to generate 3D cell spheroids. Compact multicellular spheroids with spherical morphologies were generated at seeding densities of 36 × 10^5^ and 72 × 10^5^ cells per mL (Fig. 7A). The spheroids did not show any significant changes in circularity (Fig. 7B-i) over 7 days, ranging between 0.85 and 0.94. This is likely a result of the hydrophobic and shrunken state of the microwells assisting in the maintenance of a consistent spheroid shape. Neither of the tested densities produced spheroids with circularity values below 0.85, further confirming that the spheroids maintained their structure well within these microwell arrays.

In terms of volume, a clear time-dependent pattern is evident; while 12–24 hours' incubation appears sufficient to form spheroids with relatively spherical morphologies, these spheroids then start to become compact over time, leading to a decrease in volume (Fig. 7B-ii). Integrin activity was shown to contribute to early association of cells into a loose spherical structure, followed by a delay period, then a compaction stage driven by cadherins.^60,61^ Overall, spheroid volumes displayed approx. 2- and 3.5-fold decreases after day 1 for 72- and 36 × 10^5^ cells per mL groups respectively. The higher of the two densities maintained an average spheroid volume of approx. 1.02 × 10^7^ μm^3^ between days 3–7 (down from 1.96 × 10^7^ on day 1), while the lower maintained at approx. 4.49 × 10^6^ μm^3^ in the same period (down from 1.59 × 10^7^ on day 1). Cell spheroids have previously been shown to undergo a reduction in growth rate and volume to a plateau as the necrotic core forms, and it may be likely that these spheroids are undergoing this process on day 2–3.^62–64^

In order to evaluate whether the printed microwells could support multi-layer core–shell spheroid culture, HeLa cells were added to the spheroids (red) in the microwells at half the seeding density of the HDFs (Fig. 6C, Fig. S3, ESI†). An outer layer of HeLa (green) cells was observed 24 hours post-seeding, indicating the suitability of the printed microwell arrays for generating multi-cell, multi-layer spheroids. These were observed to maintain their core–shell structure at 72 hours post-seeding as well.

### Drug release and custom structures

**Fig. 8:**
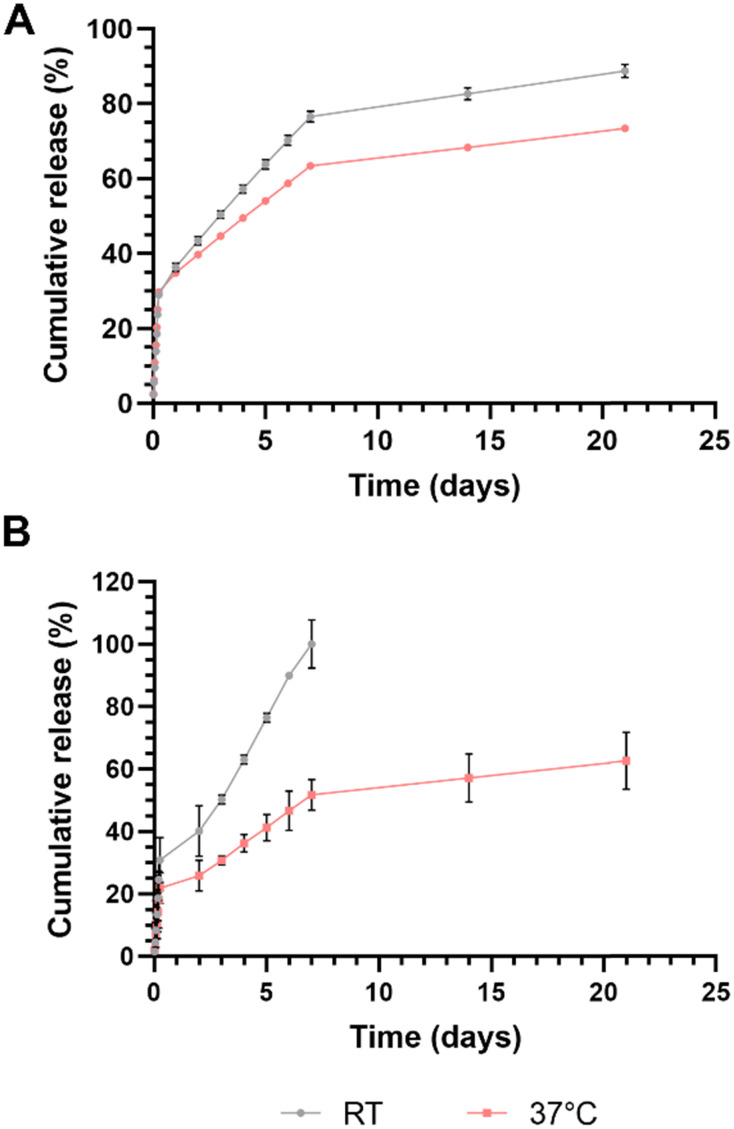
Release profiles (*n* = 3) of model drug (A) rhodamine B and (B) bovine serum albumin (BSA) from hydrogels at room temperature (RT) and 37 °C, in PBS (pH 7.4). A more extended-release profile is observed at temperatures above the formulation's LCST in both cases.

Another key application of hydrogels, particularly those with stimuli-responsive properties/capabilities is the ability for various agents, bioactive or otherwise, to be stored within them for release over time. To this end, we evaluated the release of two model drugs from within the PNIPAm hydrogel matrix printed using the final chosen formulation from the full factorial work outlined previously, in PBS at pH 7.4. Rhodamine B (479.0 Da) was chosen as a model small molecule ‘drug’, and bovine serum albumin (BSA; 66.4 kDa) was chosen as a model macromolecule ‘drug’. While BSA could be encapsulated directly within the hydrogel at the time of printing, the UV-active nature of rhodamine B meant that it would have to be encapsulated post-print through incubation in a concentrated solution of the molecule, as it would otherwise interfere with the print. This last approach is one that is frequently used in drug delivery applications associated with PNIPAm.^65,66^ Given the thermoresponsive nature of PNIPAm, a significant difference in release patterns was expected at temperatures below and above the gels’ LCST. Release of both model drugs from spherical hydrogels in PBS was thus assessed at both room temperature and at 37 °C (Fig. 8). A total of 63.4% of rhodamine B and 100.0% of BSA was found to be encapsulated within the gels.

The results of this study showed a rapid initial release of both encapsulated agents (likely from the surface and surface-adjacent volume of the gels) up to the 6-hour mark in both conditions. 28.9 ± 0.8% of encapsulated rhodamine B was released from the gels at RT in this initial phase, compared to a slightly higher 29.7 ± 0.4% at 37 °C (Fig. 8A). From this point on, the two dye release profiles diverged gradually, with gels at RT releasing higher quantities of the encapsulated dye. By day 7, there was a difference of 13.2% between the two: 76.6 ± 1.4% at RT and 63.4 ± 0.4% at 37 °C. The release pattern then slowed further, ending at 88.7 ± 1.7% of dye at RT and 73.4 ± 0.2% at 37 °C at the end of the 21-day period over which release was measured. Similarly, up to 30.8 ± 7.2% of total encapsulated BSA was released from the gels at room temperature in the burst release phase, compared to just 21.8 ± 4.9% at 37 °C (Fig. 8B). Day 2 onwards saw a rapid release of BSA at both temperatures, with spheres at RT releasing the entire remainder (69.2%) of the encapsulated protein over a 5-day period. By contrast, gels incubated at 37 °C released approximately only a further 29.9% of encapsulated BSA for a cumulative release of 51.7 ± 4.9% within the first 7 days. By the end of the 21-day period over which release was assessed, only a cumulative 62.5 ± 9.2% of BSA had been released by the gels at 37 °C.

The more rapid and complete release of both encapsulants at temperatures below the LCST is similar to results obtained by Vikulina *et al*., who noted almost complete release of entrapped fluorescein isothiocyanate-dextran (FITC-DEX) (10, 70 and 500 kDa MW) from porous PNIPAm-4-acryloylbenzophenone microgels at 22 °C and minimal release at 35 °C.^67^ They concluded that the swelling of the formulation below LCST resulted in the opening of pores, enabling faster release of encapsulated FITC-DEX while the macromolecules remained trapped within the microgels above LCST. The sharper and more rapid swelling and shrinking behavior of our hydrogels when compared to conventional chemically crosslinked PNIPAm hydrogels may have therefore contributed to the burst drug release and drug entrapment below and above LCST respectively. Cold-responsive release of encapsulated components have been observed by others using physically crosslinked PNIPAM-based nanoparticles and coatings.^68,69^ Further, a review of literature showed the existence of a specific interaction between BSA and PNIPAm at temperatures above the LCST.^70–72^ This may explain the drastically different release of the protein from the hydrogels at the different temperatures: entrapped BSA within the gels incubated at 37 °C remain tightly bound and are thus not released to completion by even day 21, ending on an even lower cumulative release than the slowest of the small molecule release curves. By contrast, identifying an underlying mechanism for the flipped release profiles for the smaller molecular weight rhodamine B is more difficult.

In either case, the formulation appears to possess a useful ability to prolong the release of molecules with wide range of size distribution at physiological pH and temperature, making these gels (and, by extension, the photoink) particularly suited for a number of *in vitro* and *in vivo* drug delivery applications by way of custom-printed drug depots, growth factor reservoirs and more. Given the ink's composition and the free radical-driven photopolymerization process involved in DLP printing, however, careful consideration must be given to the nature of the molecules employed for such release applications, and the means by which they are encapsulated within. Hydrophobic drugs are contraindicated due simply to their nature: it would be difficult to load a drug with poor aqueous solubility pre-printing due to low solubility in the photoink. Post-printing loading of hydrophobic molecules may be viable but may be affected by subsequent hydrophobic interactions with the hydrogel matrix. Drugs with disulfide groups (as one of our model drugs, BSA, possesses) may be better suited for post-printing loading, given the likelihood of thiol–ene reactions in response to radical action and/or through simple Michael addition.^73^ Loading by means of dissolution in the ink prior to printing may affect the gels’ integrity and cause less-than-optimal release due to covalent bonding of such molecules to the acrylamide groups of NIPAm- these too may be better served by post-printing loading. This may indeed be another explanation for the relatively low release of BSA seen in Fig. 8. These are all interesting avenues for future work exploring more nuanced characteristics of this photoink and the hydrogels it produces. Release profiles as a response to drug/encapsulant molecular weight, and the impact of pre-loading molecules with primary and secondary amine groups (given possible hydroamination-like reactions during the polymerization process) would be interesting to study as well.

**Fig. 9:**
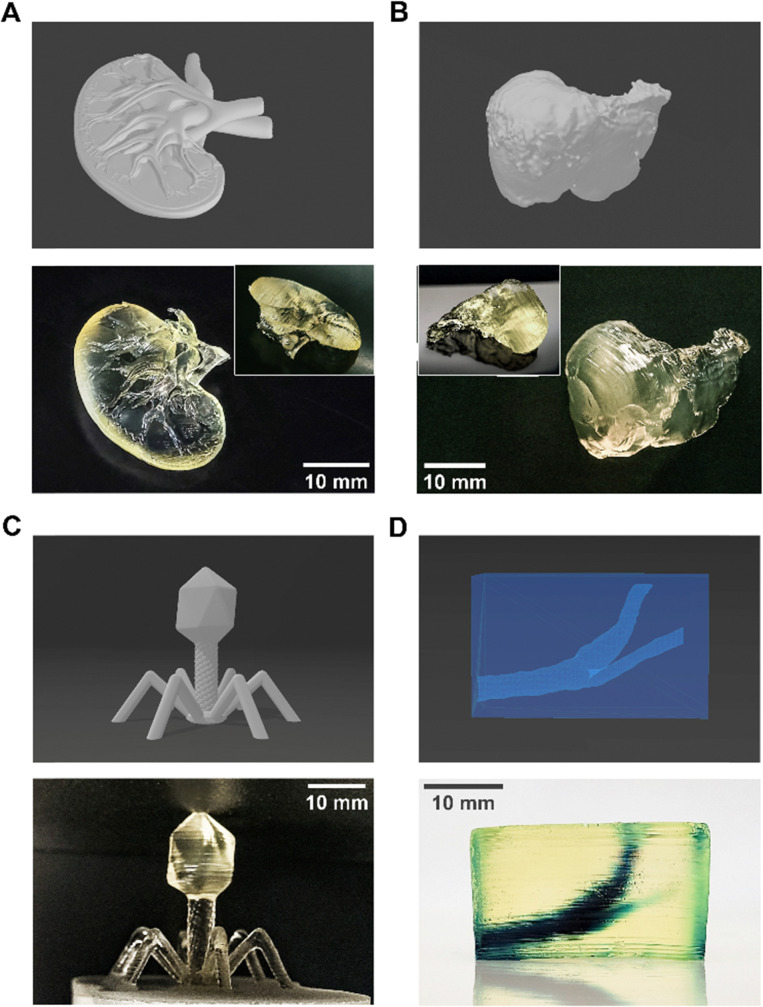
The photoink can print a range of different structures at high print fidelity – STL files *vs.* actual prints. (A) Miniature human kidney (inset: reverse); (B) miniature human liver (inset: ventral view); (C) enlarged bacteriophage virus; (D) hollow structure with human carotid (contains blue food dye for visibility; see ESI,† Video V1). All scale bars = 10 mm.

Finally, to illustrate the potential of this novel photoink, we printed several custom, high complexity (*i.e.* lacking smooth sides, exact angles, varying topography) structures, gauging the versatility of the formulation we have developed in this work particularly in the context of custom drug depots. These structures are presented in Fig. 9 and show that this photoink can print a multitude of 3D shapes at high print fidelity.

## Conclusions

This work outlined the development and optimization of a photoink formulation for DLP printing of thermoresponsive, PNIPAm-based hydrogels. Full-factorial optimization was utilized to identify a promising formulation with an LCST around that of standard culture conditions. This also revealed that high concentrations of both NIPAm and bis-acrylamide were deleterious to the printing of optically clear hydrogels, while lower concentrations yielded gels with low stiffness and strength. The hydrogels exhibited significant shrinking and swelling behaviour at temperatures above and below the LCST, respectively; these were markedly sharper than trends observed in chemically-crosslinked NIPAm gels. The ultrastructural changes that underpin these trends were characterized through the use of bulk rheology, and showed a sharp rise in storage-, elastic- and viscous moduli at the LCST, coupled with a very sharp drop in Young's modulus. The gels were found to be cytocompatible *in vitro* (>80% viability) and were thus used for the generation of both single- and multi-layer spheroids. The printed microwell arrays effectively compacted cells to form organoids with a spherical shape within 1–3 days post-seeding. The formation of multi-layer cell spheroids in particular showed the potential of this ink in tissue engineering applications. Drug release studies demonstrated the hydrogels’ ability to effectively encapsulate model drugs and release them in a temperature-dependent manner. The formulation demonstrated the ability to sustain the release of molecules with a range of sizes, making it suitable for potential *in vivo* drug delivery applications, coupled with the ability to print any structure for which a STL file could be generated.

Overall, this work highlights the successful development of a versatile photoink formulation for DLP printing of thermoresponsive hydrogels with various potential applications in tissue engineering, drug delivery, and other biomedical fields. The optimization of the formulation and the understanding of its physical properties is done in the hope that it contributes to the advancement of 3D printing technology for complex and customizable biomedical applications.

## Author contributions

K. P. and J. U. M. conceived the concept and methodology. K. P., S. B., A. V., K. M. and S. D. carried out initial optimization, sample preparation and characterization. K. P. performed data collection, curation, and visualization. K. P., S. B., K. M. and A. V. wrote the first draft of the manuscript. J. U. M. supervised the work, performed reviewing and writing/editing, and acquired funding support for the work. All authors reviewed the manuscript and approved the final version of the manuscript.

## Data availability

Most of the data supporting this article are available within the article and as part of ESI.† Due to confidentiality, any data not presented here can be made available subject to a non-disclosure agreement.

## Conflicts of interest

There are no conflicts to declare.

## Supplementary Material

TB-012-D4TB00682H-s001

TB-012-D4TB00682H-s002
