# Correction: Development and characterization of a novel poly(*N*-isopropylacrylamide)-based thermoresponsive photoink and its applications in DLP bioprinting

**DOI:** 10.1039/d4tb90152e

**Published:** 2024-09-24

**Authors:** Kalindu D. C. Perera, Sophia M. Boiani, Alexandra K. Vasta, Katherine J. Messenger, Sabrina Delva, Jyothi U. Menon

**Affiliations:** a Department of Biomedical and Pharmaceutical Sciences, College of Pharmacy, University of Rhode Island Kingston RI 02881 USA jmenon@uri.edu; b Department of Chemical Engineering, College of Engineering, University of Rhode Island Kingston RI 02881 USA; c Department of Biomedical Engineering, College of Engineering, University of Rhode Island Kingston RI 02881 USA

## Abstract

Correction for ‘Development and characterization of a novel poly(*N*-isopropylacrylamide)-based thermoresponsive photoink and its applications in DLP bioprinting’ by Kalindu D. C. Perera *et al.*, *J. Mater. Chem. B*, 2024, https://doi.org/10.1039/D4TB00682H.

The authors regret an error in the Acknowledgements section of the published article. The following funding information should be added:

S. D. was supported by the URI MARC U*STAR grant from the National Institute of General Medical Sciences of the National Institutes of Health under grant number T34GM131948.

The Royal Society of Chemistry apologises for these errors and any consequent inconvenience to authors and readers.

