# Supplementary material for: Development and characterization of a novel poly(N-isopropylacrylamide)-based thermoresponsive photoink and its applications in DLP bioprinting
Source: J Mater Chem B. 2024 Aug 29;12(38):9767–79. doi: 10.1039/d4tb00682h (PMC11373533; doi:10.1039/d4tb00682h)
Supplement: TB-012-D4TB00682H-s002 [file TB-012-D4TB00682H-s002.pdf]

Supplementary Information (SI) for Journal of Materials Chemistry B.
This journal is © The Royal Society of Chemistry 2024

Supporting Information for:

Development and Characterization of a Novel Poly(N-isopropylacrylamide)-based Thermoresponsive Photoink and its Applications in DLP Bioprinting

Kalindu D. C. Perera, Sophia M. Boiani, Alexandra K. Vasta, Katherine J. Messenger, Sabrina Delva and Jyothi U. Menon*

(*Corresponding author: jmenon@uri.edu)

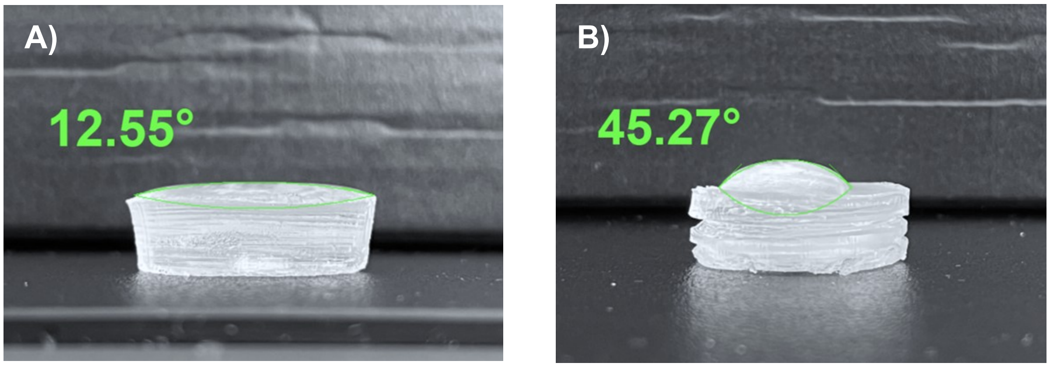

Fig. S1 Contact angle measurements for hydrogels printed using our original formulation (unchanged concentrations of NIPAm and bisacrylamide from our previous work): **A)** 4°C and **B)** 37°C.

Table S1 Formulation and results summary of full factorial experiment 1. All runs were performed in the order indicated by the statistical software, at n=2. (Bis= bisacrylamide, LAP= lithium phenyl-2,4,6-trimethylbenzoylphosphinate, LCST= lower critical solution temperature, Y/N= yes/no).

Run#	[NIPAm] (mg/mL)	[Bis] (mg/mL)	[LAP] (mg/mL)	[Tartrazine] (mg/mL)	Bleaching	LCST (°C)
1	100	4	2	0.25	N	34.9
2	200	8	2	0.1	Y	-
3	100	4	1	0.25	N	33.3
4	200	4	2	0.25	N	40.1
5	200	4	1	0.1	Y	-
6	200	4	2	0.1	Y	-
7	100	8	2	0.1	N	33.1
8	100	4	1	0.1	N	32.2
9	100	8	1	0.1	N	32.9
10	200	4	1	0.25	N	29.7
11	200	8	1	0.1	Y	-
12	100	4	2	0.1	N	34.7
13	100	8	2	0.25	N	32.9
14	200	8	2	0.25	Y	-
15	100	8	1	0.25	N	31.2
16	200	8	1	0.25	Y	-

Table S2 Parameter and results summary of full factorial experiment 2. All runs were performed in the order indicated by the statistical software, at n=2. Highlighted in grey are runs that were not completed (see Results & Discussion).

Run#	Power (%)	Exposure time (s)	Base layer exposure factor (x)
1	44	20	4
2	36	20	4
3	44	16	2
4	36	20	4
5	44	16	4
6	44	16	4
7	36	20	2
8	36	16	2
9	36	16	2
10	36	16	4
11	44	20	2
12	44	16	2
13	36	16	4
14	44	20	2
15	44	20	4
16	36	20	2

Table S3 Formulation and results summary of full factorial experiment 3. All runs were performed in the order indicated by the statistical software, at n=2. (Bis= bisacrylamide, LAP= lithium phenyl-2,4,6-trimethylbenzoylphosphinate, LCST= lower critical solution temperature, Y/N= yes/no). Highlighted in grey is the formulation selected as final for all characterization.

Run#	[NIPAm] (mg/mL)	[Bis] (mg/mL)	[LAP] (mg/mL)	[Tartrazine] (mg/mL)	Bleaching	LCST (°C)
1	100	16	2	0.5	Y	-
2	150	16	2	0.5	Y	-
3	100	8	3	0.5	N	32.1
4	100	16	3	0.5	Y	-
5	100	8	2	0.25	N	34.7
6	150	16	3	0.25	Y	-
7	150	8	2	0.5	N	36.8
8	150	16	2	0.25	Y	-
9	100	8	2	0.5	Y	-
10	150	16	3	0.5	Y	-
11	100	8	3	0.25	N	30.7
12	150	8	3	0.5	Y	-
13	150	8	2	0.25	N	33.8
14	100	16	3	0.25	Y	-
15	100	16	2	0.25	Y	-
16	150	8	4	0.25	N	40.3

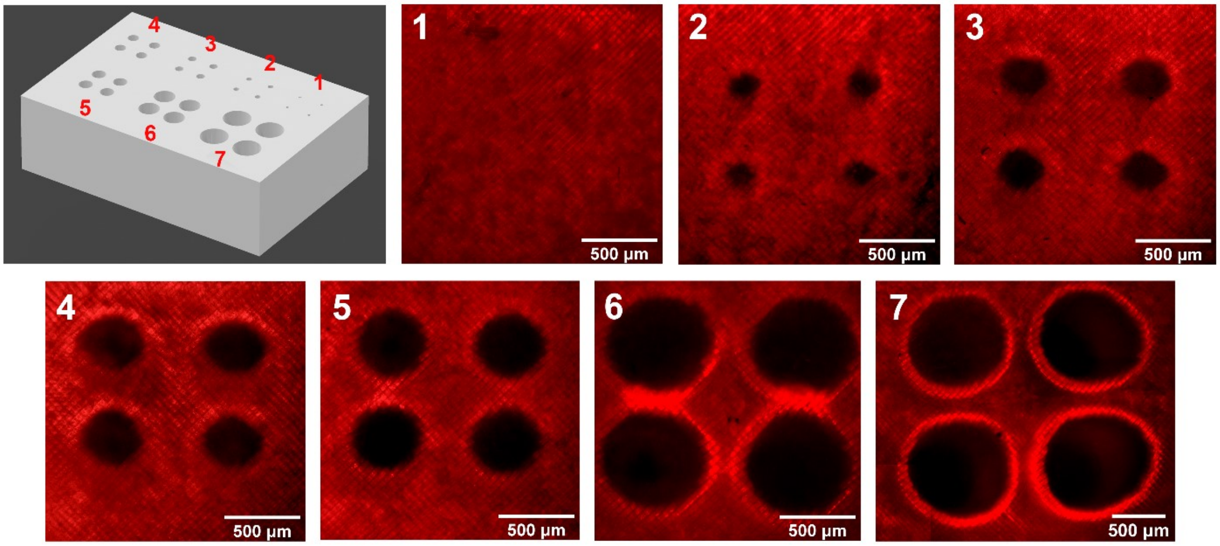

Fig. S2 EVOS fluorescence micrographs of hydrogel with channels of increasing diameter (1= 100 µm; 2= 200 µm; 3= 300 µm; 4= 400 µm; 5= 500 µm; 6= 750 µm, 7= 1000 µm). Gel loaded with rhodamine B for increased contrast. Print limit for circular features is 200 µm: note absence of 100 µm channels. All scale bars= 200 µm. (Note: micrograph of 1000 µm channels is a stitched composite, owing to size of wells and microscope's limited imaging field).

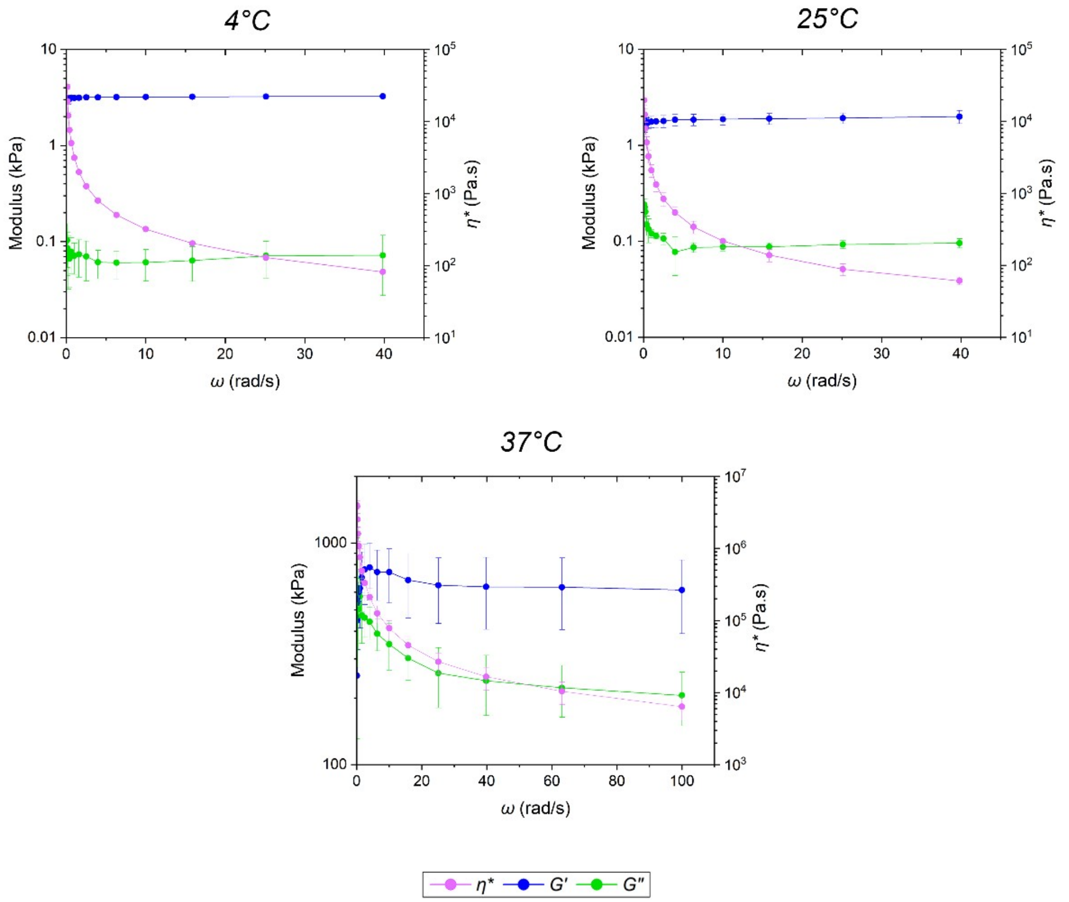

Fig. S3 Mean trends of rheological parameters of PNIPAm discs at different temperatures.

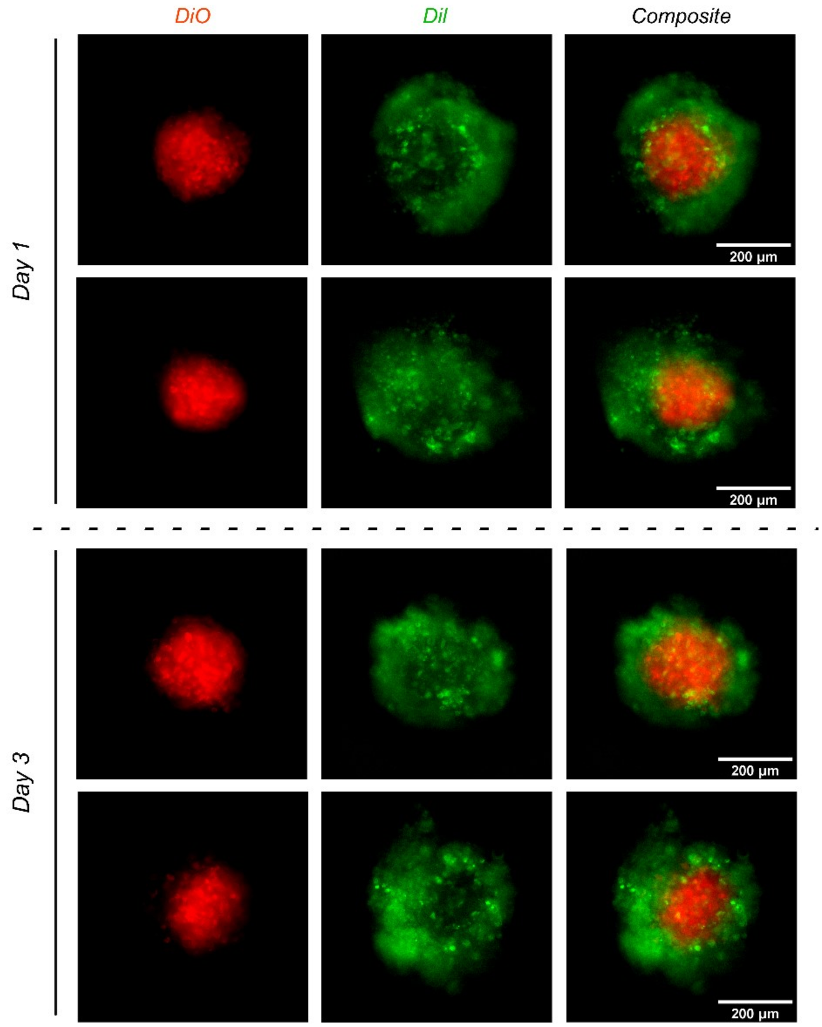

Fig. S4 EVOS fluorescence micrographs of bilayer spheroids, days 1 and 3 (red/inner= HDFs, green/outer= HeLa). All scale bars= 200 µm.
